# The Critical Role of *AtPAP17* and *AtPAP26* Genes in Arabidopsis Phosphate Compensation Network

**DOI:** 10.3389/fpls.2020.565865

**Published:** 2020-09-30

**Authors:** Siamak Farhadi, Mohammad Sadegh Sabet, Mohammad Ali Malboobi, Ahmad Moieni

**Affiliations:** ^1^ Department of Plant Genetics and Breeding, Faculty of Agriculture, Tarbiat Modares University, Tehran, Iran; ^2^ Department of Plant Biotechnology, National Institute of Genetic Engineering and Biotechnology, Tehran, Iran

**Keywords:** transcriptional adaptation, phosphorus, purple acid phosphatase, genetic interaction, double mutant plant, *AtPAP17*, *AtPAP26*

## Abstract

Purple acid phosphatases (*PAP*)-encoding genes form a complex network that play a critical role in plant phosphate (Pi) homeostasis. Mostly, the functions of PAPs were investigated individually. However, the interactions of most of these genes in response to various concentrations of available Pi remain unknown. In this study, the roles of *AtPAP17* and *AtPAP26* genes, and their relationship within Pi homeostasis context were investigated. Surprisingly, *atpap17* and *atpap26* mutants not only showed no obvious developmental defects, but also produced higher biomass in compare to wild type (WT) plants under normal growth conditions. Comparing gene expression patterns of these mutants with WT plant, we identified a set of genes up-regulated in mutant plants but not in WT. Based on these unexpected results and up-regulation of *AtPAP17* and *AtPAP26* genes by the loss of function of each other, the hypothesis of compensation relationship between these genes in Pi homeostasis was assessed by generating *atpap17/atpap26* double mutants. Observation of developmental defects in *atpap17/atpap26* mutant but not in single mutants indicated a compensation relationship between *AtPAP17* and *AtPAP26* genes in Pi homeostasis network. Taken together, these results demonstrate the activation of *AtPAP17* and *AtPAP26* genes to buffer against the loss of function of each other, and this compensation relationship is vital for Arabidopsis growth and development.

## Introduction

Phosphorus (P) is one of the critical nutrients required for plant growth and development. This element plays a key role in the pivotal processes including photosynthesis, energy generation, as well as all cellular phosphorylation events (Raghothama, 1999; Ticconi and Abel, 2004; Huang et al., 2008; Tran et al., 2010a). In addition, Pi is a constituent of the major molecules including phospholipids, nucleic acids, and ATP (Poirier and Bucher, 2002; Tran et al., 2010a; Péret et al., 2011). However, the availability of soluble Pi in most agricultural lands is very low, so that it is a common abiotic stress that limits plant growth and production (Plaxton and Tran, 2011; López-Arredondo et al., 2014). Phosphate reserves in soil are mostly in the form of organic, which cannot be absorbed by plants (Richardson, 2009; Shen et al., 2011; Stutter et al., 2012). To overcome low Pi availability, plants have evolved several response mechanisms for increasing Pi availability and maintaining its homeostasis that are collectively known as the Pi-starvation response (PSR) (Vance et al., 2003; Hammond et al., 2004; Ticconi and Abel, 2004; Yuan and Liu, 2008; Fang et al., 2009; Tran et al., 2010a; Plaxton and Tran, 2011). The induction and secretion of acid phosphatases (APases) is one of the plant responses to Pi deficiency that catalyze the hydrolysis of orthophosphate from a wide range of phosphoesters and anhydrides under acidic conditions (Duff et al., 1994; Tran et al., 2010a; Song and Liu, 2015; Tian and Liao, 2015). APases are involved in the scavenging, releasing, and recycling of Pi, particularly when it is limited (Duff et al., 1994; Robinson et al., 2012b). APases can be assigned to two groups including extracellular (secreted) and intracellular APases (Tian et al., 2012; Tian and Liao, 2015). Pi starvation inducible (PSI) extracellular APases are involved in Pi scavenging and hydrolysis from external phosphoesters or organic P, whereas intracellular APases remobilize and recycle Pi from its compound in cytoplasm and organelles (Veljanovski et al., 2006; Tran et al., 2010a; Wang et al., 2014; Zhang et al., 2014; Wang and Liu, 2018). Several PSI APases have been biochemically and molecularly characterized in some plant species such as *Arabidopsis thaliana* (Veljanovski et al., 2006; Lohrasebi et al., 2007; Kuang et al., 2009; Tran et al., 2010b; Wang et al., 2011; Zamani et al., 2014; Liu et al., 2016; Bhadouria et al., 2017; Kong et al., 2018; Sabet et al., 2018). Among APases, purple acid phosphatases (PAPs) are the most important class of plant PSI APases which appear purple or pink color in water solution due to a bimetallic active center (Olczak et al., 2003; Tran et al., 2010a). According to genome sequence analysis and annotation, this gene family, PAPs, includes 29 members in *Arabidopsis thaliana* (AtPAPs), which several of them are induced by Pi deprivation (Del Pozo et al., 1999; Haran et al., 2000; Li et al., 2002; Tran et al., 2010a; Wang et al., 2011). Arabidopsis PAP family has been categorized into high and low molecular weight phosphatases based on their deduced amino acid sequences (Li et al., 2002). Recent biochemical and functional genomic studies confirm the effects of several PAPs, particularly *AtPAP26* and *AtPAP17*, on the utilization and mobilization of intracellular or extracellular Pi in Arabidopsis (Veljanovski et al., 2006; Hurley et al., 2010; Wang et al., 2011; Robinson et al., 2012b; Wang et al., 2014). In addition, some PAPs including AtPAP26 and AtPAP17 have been induced markedly during leaf senescence and remobilize Pi from senescing leaves. AtPAP26 belongs to high molecular weight PAPs, with a molecular mass of ~55 kDa/monomer, and is closely related to other key PAPs such as AtPAP10 and AtPAP12, whereas AtPAP17 as a low molecular weight PAP is more closely related to mammalian PAPs and has a molecular mass of ~35 kDa (Del Pozo et al., 1999; Haran et al., 2000; Li et al., 2002; Tran et al., 2010b). The analysis of an *atpap26* T-DNA insertion mutant showed that *AtPAP26* gene is a dominant contributor to intracellular APase activity (Hurley et al., 2010). *AtPAP26* is also known as only dual-targeted PAP, which is the major intracellular (vacuolar) as well as secreted APase up-regulated by Pi deficient conditions in Arabidopsis (Hurley et al., 2010). The studies showed that *AtPAP26* has a significant contribution in Pi metabolism of *Arabidopsis thaliana* (Veljanovski et al., 2006; Hurley et al., 2010; Tran et al., 2010b; Robinson et al., 2012a; Robinson et al., 2012b). *AtPAP17* is another member of Arabidopsis PAP family that markedly induced by Pi-starvation and it was the first PSI PAP characterized in Arabidopsis under Pi deficient conditions (Del Pozo et al., 1999). However, its subcellular localization and biological function(s) partly remain unknown. *AtPAP17* and *AtPAP26* genes were previously investigated individually. However, the interaction of these genes in response to Pi limitation has not been assessed yet. To date, it is well known that a complex relationship exists among genes related to Pi hemostasis network because of the importance of maintaining Pi homeostasis in the cell (Rouached et al., 2010; Liang et al., 2014). Therefore, studying the interaction of APase-encoding genes under different Pi concentrations is essential for better understanding the role of these genes in providing plant required Pi. For this purpose, due to the complex network and coordination regulation between these genes, generation of single and double or multiple mutant lines of phosphatase genes is essential. In this study, the interactions between two important members of PAP family, *AtPAP17* and *AtPAP26* genes, in Pi complex gene network were investigated for the first time.

## Materials and Methods

### Plant Material and Growth Conditions

*Arabidopsis thaliana* ecotype Columbia (Col-0) was used for all experiments in this study. For mutants isolation, Arabidopsis seeds were planted in soil mixture “peat moss, perlite and vermiculite with 1:1:1 ratio,” then the seeds were stratified at 4°C for 2 days. Afterwards, they were placed in growth chamber with a 16-h-light/8-h-dark photoperiod at 25°C and fertilized twice weekly by sub-irrigation with Hoagland’s nutrient solution. Also, transformed Arabidopsis plants carrying *CaMV-35S:AtPAP17* and *CaMV-35S:AtPAP26* constructs were used as overexpressing lines of *AtPAP17* and *AtPAP26* genes, respectively (Sabet et al., 2018). More than 10 single-copy transgenic lines were generated for each construct. Finally, four independent transgenic lines were used for overexpression studies of *AtPAP17* and *AtPAP26* (**Supplementary Figure S1**).

To investigate the morphological, physiological, and molecular changes of plants, seeds were surface-sterilized, stratified, and placed in plates containing solidified full-strength MS medium (Murashige and Skoog, 1962), pH = 5.8, with 1.25 mM KH_2_PO_4_ and supplemented with 1% (w/v) sucrose and 0.7% (w/v) plant-agar. One week after seed germination, 7-day-old seedlings were transplanted into fresh media containing filter-sterilized 1.25 mM KH_2_PO_4_ (Pi-sufficient medium) and no Pi (Pi-deficient medium) and placed in growth chambers as described above for 14 days. For Pi-deficient medium (referred as −Pi), KH_2_PO_4_ in +Pi medium was replaced by 1.25 mM K_2_SO_4_. The seedlings from both conditions (−Pi and +Pi) were harvested at day 21, then the shoots and roots were rapidly excised, frozen in liquid N_2_ and stored at −80°C.

### Generation of a*tpap17/atpap26* Double-Knockout Mutant

Two *atpap17* and *atpap26* T-DNA insertion mutants (SALK-097940.47.75.x and SALK-152821, respectively) were used in this study. Homozygous mutant plants carrying T-DNA insertions in both alleles of each gene, were isolated by PCR-screening using T-DNA left-border and gene-specific primers. To generate *atpap17/atpap26* double mutant line, *atpap17* mutant line (pollen donor) was crossed into *atpap26* plants (pollen receptor). The presence of T-DNA insertion in both *AtPAP17* and *AtPAP26* genes of F_1_ plants was verified by PCR-screening. F_1_ plants were self-pollinated, and homozygous *atpap17/atpap26* double mutants were identified by PCR-screening of F_2_ progenies using a T-DNA left-border and *AtPAP17*- and *AtPAP26*-specific primers (**Figure 2C** and **Supplementary Table S1**).

### Protein Extraction and APase Assays

Acid phosphatase activity was determined by hydrolysis of *ρ*-nitrophenylphosphate (*ρ*NPP) to *ρ*NP and Pi. For quantitative analysis of APase activity in shoot and root tissues, 50 mg of samples were ground to fine powder in liquid nitrogen and homogenized in ice-cold extraction buffer (100 mM sodium acetate, pH 5.6). Homogenates were centrifuged twice at 14,000 g for 10 min at 4°C, and the supernatants were used for enzyme assays (Naseri et al., 2004). APase activity was assayed at 37°C for 30 min in 100 mM sodium acetate buffer (pH = 5.6) containing 5 mM *p*NPP. Released Pi was measured spectrophotometrically by adding AAM buffer containing acetone, 5 N sulfuric acid, and 10 mM ammonium molybdate (2:1:1 by vol.). After stopping the reaction by adding citric acid (1 M), absorbance was read at 355 nm. Protein concentrations were determined by the Bradford assay (Bradford, 1976) using bovine serum albumin as the standard.

### Quantitative Analysis of Cellular P Content

The analysis of cellular P content was carried out using a modified assay of Ames (Ames, 1966). For the quantification of total P content, 10% nitrate magnesium (w/v in 95% ethanol) was added to 50 mg fresh tissues to final volume of 1.5 ml in a Pyrex tube. The biomaterial was flamed to ash by heating the tubes over the strong flame until vanish the brown fumes. After cooling the tubes, 500 μl of concentrated perchloric acid was added to the ash and incubated at room temperature for 24 h. For hydrolyzing any pyrophosphate formed in the ash, the tubes were heated in a boiling water bath for 60 min and the final volume was brought to 2 ml by distilled water. About 50 μl of the supernatant was mixed with 250 μl of distilled water and 700 μl of Pi assay reagent (0.42% ammonium molybdate in 1 N sulfuric acid and 10% ascorbic acid with a 6:1 mix ratio). The reaction was incubated at 45°C for 20 min and P content was determined at A_820_ according to a standard calibration curve and expressed as micromoles of P/g fresh weight (FW).

### Microarray Data Analysis

Microarray data from GEO database (GSE33790) (Woo et al., 2012) were used as a primary means of evaluating the expression of Arabidopsis PAP genes in response to different Pi conditions. Alterations in the expression level of PAP genes were investigated in Arabidopsis seedlings and roots in response to Pi-starved and re-fed conditions.

### RNA Extraction and Gene Expression Analysis

Total RNA was extracted from shoot and root of 21-day-old seedlings using RNX-plus RNA purification kit (CinnaGen, Tehran, I.R. Iran) according to the manufacturer’s manual. Genomic DNA contamination in extracted RNA samples was removed using RNase-free-DNase-I (Fermentas, Life Sciences, ON, Canada). For cDNA synthesis, 1 µg of DNase-treated RNA was reverse transcribed by M-MLV reverse transcriptase (Fermentas, Lithuania) according to the manufacturer’s instructions. Quantitative real time-PCR was carried out with SYBR green fluorescent dye in an ABI StepOnePlus™ real time-PCR system (Applied Biosystems) using gene specific primers (**Supplementary Table S1**). PCR amplification was performed under following conditions: 95°C for 3 min, followed by 40 cycles at 95°C for 20 s, 58°C for 20 s, and 72°C for 20 s, and a final step at 72°C for 5 min. Semi-quantitative RT-PCR was performed as described previously (Zamani et al., 2012; Sabet et al., 2018). Also, *actin* and α-*tubulin* genes were used as internal controls to normalize gene expression in quantitative real time-PCR and semi-quantitative RT-PCR, respectively. All PCR reactions were performed in three biological replicates. Relative expression levels of all genes were calculated using 2^−ΔCT^ method (Livak and Schmittgen, 2001). All the genes and primers used for RT-PCR are listed in **Supplementary Table S1**.

### Statistical Analysis

All assays were carried out as factorial experiment based on a randomized complete block design (RCBD). All values are represented as an average of three biological replicates ± SE. Data were analyzed using ANOVA and means comparison was conducted by least significant difference (LSD) using SAS (SAS 9.0) and SPSS (SPSS 22.0). Also *p*-value <0.05 was assumed for significant differences. GraphPad Prism 7 software was used to plot statistical graphs.

## Results

### AtPAPs Expression Pattern in Response to Pi Starvation

**Figure 1:**
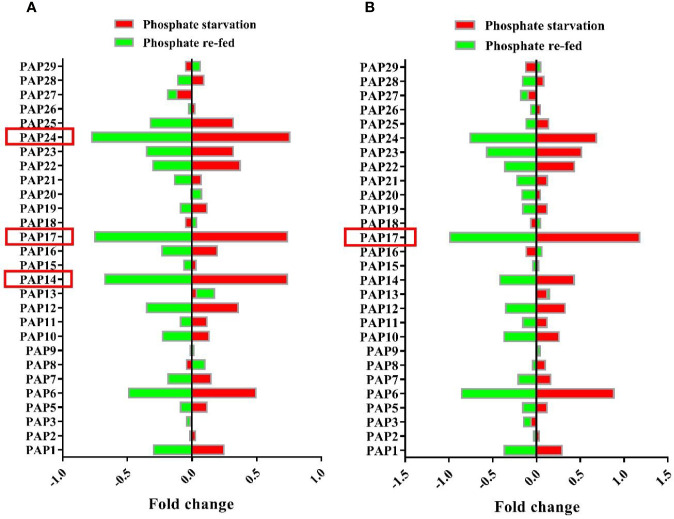
Microarray analysis of gene expression patterns of Arabidopsis PAP family members under Pi starvation and re-fed in seedlings **(A)** and roots **(B)** of Arabidopsis plant. Plants were grown hydroponically for 10 days in Pi-sufficient (1.75 mM Na_3_PO_4_) or Pi-deficient (no Na_3_PO_4_) conditions. For Pi re-fed treatment, plants were grown for 10 days under Pi-deficient (no Na_3_PO_4_) conditions and then transferred to +Pi (1.75 mM Na_3_PO_4_) medium for 3 days. Values are the means of fold changes in PAP-encoding genes expression of three biological replicates.

The maintenance of Pi homeostasis is governed by multidimensional gene regulation networks in plants (Liang et al., 2014). *PAP*s are one of the gene families in Pi complex gene network that play a critical role in Pi homeostasis. In this study, the microarray data from GEO database (GSE33790; Woo et al., 2012) were used to inspect AtPAP genes expression in response to Pi deficiency. The analysis of microarray gene expression data showed that among this gene family, the expression levels of *AtPAP14*, *AtPAP17*, and *AtPAP24* genes were markedly induced by phosphate starvation (**Figure 1A**). Also, analysis of root microarray gene expression data showed that the most gene expression change in response to Pi starvation and Pi re-fed is related to *AtPAP17* (**Figure 1B**). In contrast, some PAPs constitutively expressed under both Pi starvation and Pi re-fed conditions.

### Identification of *atpap17* and *atpap26* Single and Double Mutants

**Figure 2:**
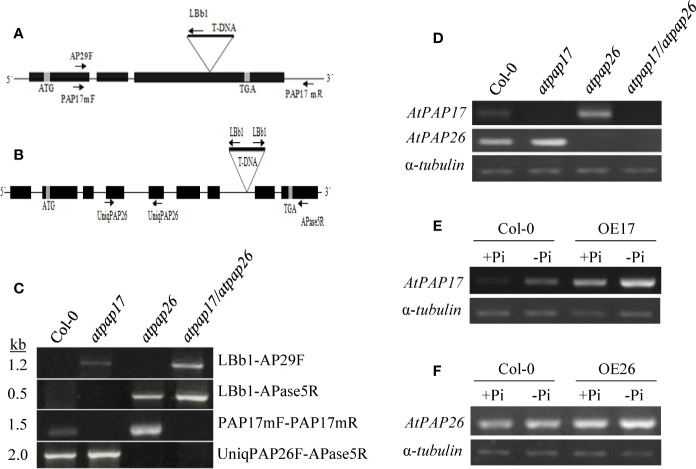
Schematic representation of *AtPAP17* **(A)** and *AtPAP26* **(B)** genes; solid boxes and lines represent exons and introns, respectively. Evaluation and verification of homozygosity of mutants *via* PCR-based screening of gDNA using T-DNA left-border and *AtPAP17* and *AtPAP26*-specific primers **(C)**. The T-DNA insertion location is indicated by “triangles-T-DNA,” and arrows represent primers used for PCR and genotyping. Levels of *AtPAP17* and *AtPAP26* transcripts were quantified by semi-qRT-PCR in single and double mutants **(D)**. Semi-quantitative RT-PCR of *AtPAP17* **(E)** and *AtPAP26* **(F)** expression in homozygous *AtPAP17*- and *AtPAP26*-overexpressed lines (OE17 and OE26), respectively, grown under +Pi (1.25 mM KH_2_PO_4_) and −Pi (no Pi) conditions. Also, α-tubulin was used as an internal control for normalization.

Reverse genetics approaches such as gene knockout and overexpression of a specific gene are useful for investigating the gene functional assignments (Alonso et al., 2003). In this study, two T-DNA insertion lines “SALK_097940.47.75.x and Salk_152821” were used to reveal the function of *AtPAP17* and *AtPAP26* genes, respectively. T-DNA insertions in *atpap17* and *atpap26* mutant genome were predicted to be occurred in the third exon of *AtPAP17* (locus At3g17790) and the seventh intron of *AtPAP26* (locus At5g34850) genes, respectively (**Figures 2A, B** **)**. These positions were confirmed by PCR screening of gDNA using T-DNA left-border and *AtPAP17*- and *AtPAP26*-specific primers (**Figure 2C**). Also, the homozygosity of T-DNA mutants was verified by PCR on genomic DNA using *AtPAP17*- and *AtPAP26*-specific primers (**Figure 2C**).

These homozygous single mutant lines were crossed to generate *atpap17/atpap26* double mutant line. Finally, the loss of *AtPAP17* and *AtPAP26* genes expression in homozygous *atpap17* and *atpap26* single mutant lines, respectively, and also loss of expression of both *AtPAP17* and *AtPAP26* genes in *atpap17/atpap26* double mutant line were verified by semi-quantitative RT-PCR of cDNA using *AtPAP17*- and *AtPAP26*-specific primers (**Figure 2D**).

### Relationship Between *AtPAP17* and Other PAP-Encoding Genes

**Figure 3:**
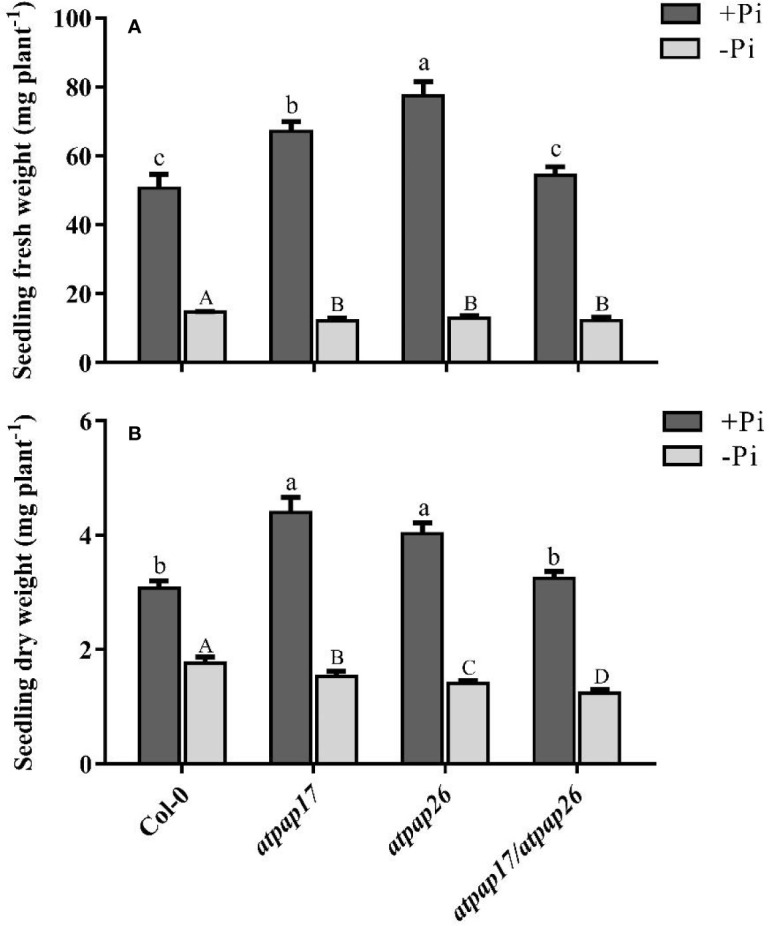
Seedling fresh weight **(A)** and dry weight **(B)** of Col-0, *AtPAP17* and *AtPAP26* mutants (*atpap17*, *atpap26*, and *atpap17*/*atpap26*). Plants grown for 7 days under +Pi (1.25 mM KH_2_PO_4_) then transferred to +Pi (1.25 mM KH_2_PO_4_) and −Pi (no Pi) conditions for 14 days as described in the materials and methods. Values are the means ± SE of three biological replicates. Significant differences are indicated by different letters above the bars (*P* < 0.05).

**Figure 4:**
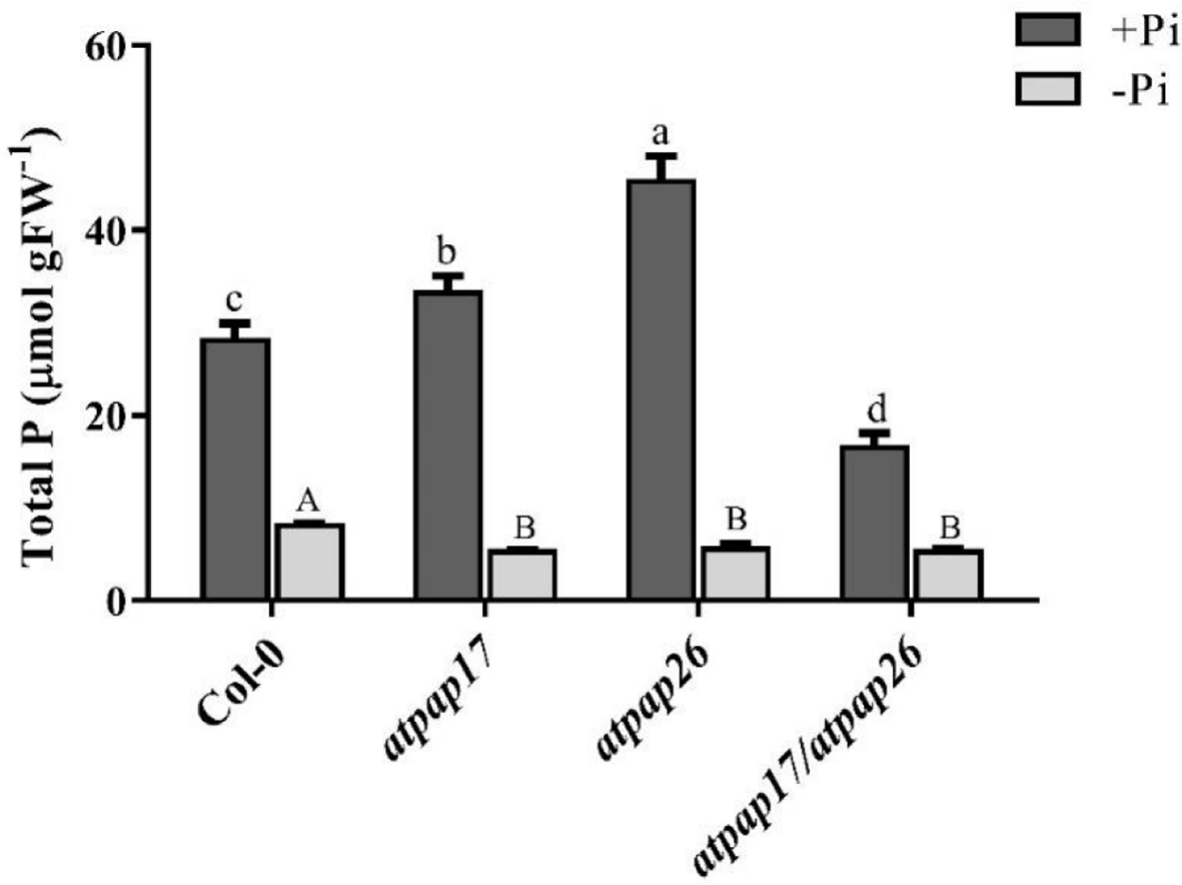
Total P content of 21-day-old Arabidopsis seedlings of Col-0, *AtPAP17* and *AtPAP26* mutants (*atpap17*, *atpap26*, and *atpap17/atpap26*) grown under +Pi (1.25 mM KH_2_PO_4_) and −Pi (no Pi) conditions. Values are the means ± SE of three biological replicates. Significant differences are indicated by different letters above the bars (*P* < 0.05).

To assess the effect of *AtPAP17* gene on plant growth and development, homozygous T-DNA insertion mutant line “*atpap17”* was grown on Pi-sufficient (+Pi) and Pi-deficient (−Pi) media. Unexpectedly the knockout of *AtPAP17* gene was accompanied by 33% increase in fresh weight (FW) of 21-day-old *atpap17* seedlings as compared to Col-0 under +Pi conditions (**Figure 3A**). Also, the knockout of *AtPAP17* gene resulted in a significant increase in *atpap17* mutant dry weight (DW) (about 43% relative to Col-0) under Pi-sufficient conditions (**Figure 3B**). Additionally, P content was significantly increased (about 18% relative to Col-0) by *AtPAP17* gene destruction under +Pi conditions (**Figure 4**).

**Figure 5:**
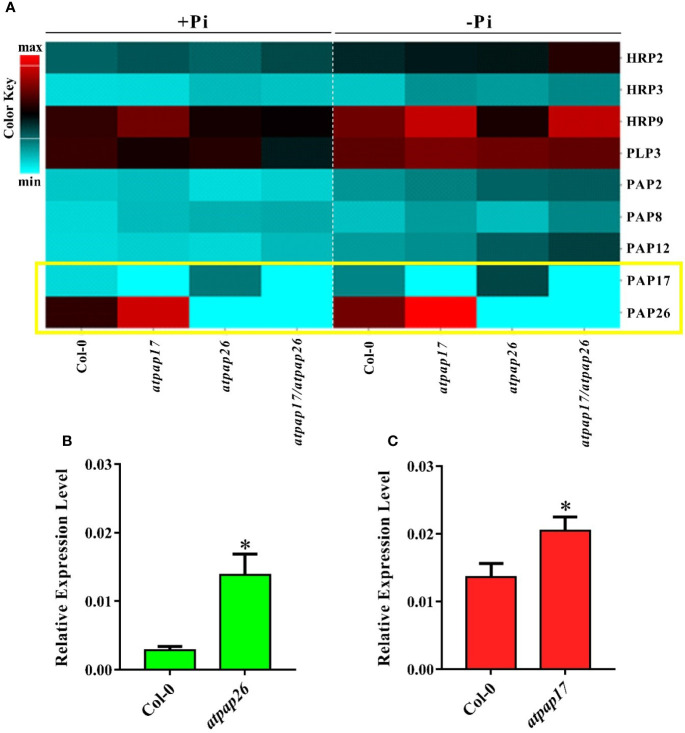
Heat map of expression pattern of some APase genes in Col-0, *atpap17*, *atpap26*, and *atpap17/atpap26* mutants grown under Pi-sufficient (+Pi, 1.25 mM KH_2_PO_4_) and −Pi (no Pi) conditions **(A)**. Real time PCR analysis of the expression of *AtPAP17* **(B)** and *AtPAP26* **(C)** in +Pi seedling of Col-0, *atpap26* and *atpap17* mutants. Total RNA was extracted from 21-day-old plants with three biological replicates. Asterisks (*) indicate values that are significantly different from that of the WT.

Based on postulated gene networking for regulation of Pi homeostasis in plant cells (Misson et al., 2005; Rouached et al., 2010), the possibility of alterations in expression levels of some APase-encoding genes related to Pi homeostasis was inspected under Pi-sufficient conditions. The results showed that *AtPAP17* gene knockout resulted in the significant up-regulation of some APase genes including *HRP9*, *AtPAP8*, and *AtPAP26* in the seedling of *atpap17* single mutant (**Figures 5A, C** **)**. These results clearly demonstrated that the highest difference in expression levels of the studied genes occurred for *AtPAP26* gene in *atpap17* mutant line (**Figure 5A**).

According to notable induction of *AtPAP17* activity in response to Pi starvation (**Figures 1**, **5**) and the high expression level of *AtPAP26* gene in *atpap17* mutant plants, it seems that a strong relationship between *AtPAP17* and *AtPAP26* genes could be involved in Pi homeostasis in plant cells. Therefore, an *AtPAP26* T-DNA insertion mutant, *atpap26*, was used to examine this hypothesis. In the same way, the unexpected increase in DW and P content (about 32 and 62% relative to Col-0, respectively) was observed in *atpap26* mutant grown under +Pi conditions (**Figures 3B**, **4**). Also, the seedlings FW of 21-day-old *atpap26* lines grown under +Pi conditions significantly increased by about 54% relative to Col-0 (**Figure 3A**). The complementation of the homozygous lines of *atpap17* and *atpap26* mutants with *AtPAP17* and *AtPAP26* genes, respectively, abolished phenotypes observed in these mutants showing the compensation of functions of the respective genes (data not shown). The expression patterns of Arabidopsis APase genes in response to *AtPAP26* gene destruction revealed that the expression levels of *AtPAP17*, *AtPAP8*, and *HRP3* genes increased in *atpap26* mutant grown under +Pi conditions (**Figures 5A, B** **)**. The considerable increase in the expression levels of *AtPAP17* and *AtPAP26* genes in *atpap26* and *atpap17* mutants, respectively, suggested reciprocal relationship between *AtPAP17* and *AtPAP26* genes in Pi homeostasis. This relationship was evaluated by simultaneous destruction of these two genes using T-DNA insertion.

### Double Mutant Lines “*atpap17/atpap26*” Illustrated the Relationship Between *AtPAP17* and *AtPAP26* Genes

To study the effect of simultaneous destruction of *AtPAP17* and *AtPAP26* genes on plant growth and P content, *atpap17/atpap26* line was grown in Pi-sufficient and Pi-deficient conditions. Our results revealed that seedling DW of *atpap17/atpap26* double mutant line was lower than that of *atpap17* and *atpap26* single mutants in both +Pi and −Pi conditions. DW of *atpap17/atpap26* line was 27 and 21% lower than that of *atpap17* and *atpap26* single mutant lines, respectively, under +Pi conditions (**Figure 3B**). Under −Pi conditions, DW significantly decreased in *atpap17/atpap26* double mutant line as compared to *atpap17* and *atpap26* single mutant lines by about 23 and 13%, respectively (**Figure 3B**). Our results showed that simultaneous destruction of these two genes markedly reduced P content in *atpap17/atpap26* double mutant line (**Figure 4**). So that, P content of *atpap17/atpap26* double mutant line was 51 and 64% lower than that of *atpap17* and *atpap26* single mutant lines under +Pi conditions (**Figure 4**). These results clearly showed a compensation relationship between *AtPAP17* and *AtPAP26* genes in Pi homeostasis. Besides, simultaneous destruction of *AtPAP17* and *AtPAP26* genes led to the up-regulation of *HRP2*, *HRP3*, *AtPAP8*, and *AtPAP12* genes in seedlings of *atpap17/atpap26* double mutant line under +Pi conditions. In contrast, *HRP2*, *HRP3*, *HRP9*, *AtPAP2*, *AtPAP8*, and *AtPAP12* genes were up-regulated in *atpap17/atpap26* double mutant line under Pi starvation (**Figure 5A**).

Our results showed that P content in all mutant lines were significantly decreased as compared to Col-0 under Pi starvation (**Figure 4**). In addition, seedlings FW of mutant lines was lower than that of Col-0 under −Pi conditions, while no significant differences in seedlings FW were found among mutant lines (**Figure 3A**). The study of gene expression pattern under −Pi conditions indicated that *AtPAP17* gene knockout was accompanied with the up-regulation of *HRP3*, *HRP9*, *AtPAP8*, and *AtPAP26* genes. Meanwhile, *HRP3*, *AtPAP2*, *AtPAP12*, and *AtPAP17* genes were up-regulated in *atpap26* mutant line under Pi starvation (**Figure 5A**).

### Acid Phosphatase Activity

**Figure 6:**
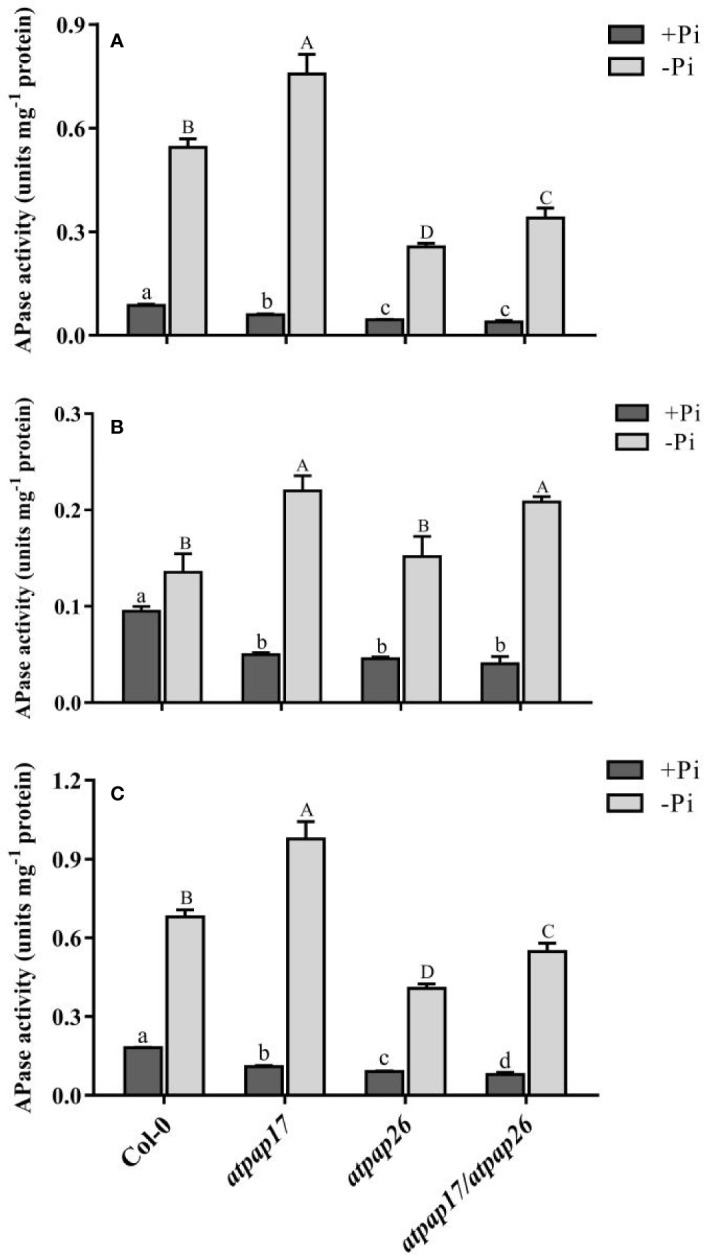
APase activities of Col-0, *AtPAP17* and *AtPAP26* mutants (*atpap17*, *atpap26*, and *atpap17/atpap26*) grown under +Pi (1.25 mM KH_2_PO_4_) and −Pi (no Pi) conditions. APase activities of shoot **(A)**, root **(B)**, and seedling **(C)** of the lines as determined with *p*NPP as the substrate. Values are the means ± SE of three biological replicates. Significant differences are indicated by different letters above the bars (*P* < 0.05).

To study the relative contributions of *AtPAP17* and *AtPAP26* genes to total intracellular phosphatase activity, APase activity of T-DNA insertion mutant lines were assayed under both +Pi and −Pi conditions. Analysis of APase activity against *p*NPP, a general substrate, indicated that total APase activity of all plants grown under −Pi conditions was significantly higher than that of the plants grown under +Pi conditions. APase activity in the seedlings of *atpap17*, *atpap26*, and *atpap17/atpap26* lines significantly decreased by about 40, 50, and 56% as compared to Col-0 under Pi-sufficient conditions, respectively (**Figure 6C**). The seedling APase activity of *atpap17/atpap26* double mutant line was by about 28 and 13% lower than that of *atpap17* and *atpap26* single mutant lines, respectively, under the same conditions (**Figure 6C**). In contrast, APase activity in the seedling of *atpap17* line was 43% higher than that of Col-0 under −Pi conditions. However, APase activity in seedling of *atpap26* and *atpap17/atpap26* lines was about 40 and 19% lower than that of Col-0, respectively, under Pi-deficient conditions (**Figure 6C**).

APase activities were also investigated separately in the shoots and roots of mutant lines grown under +Pi and −Pi conditions. APase activity was reduced by destruction of *AtPAP17* and *AtPAP26* genes in the shoots and roots as compared to Col-0 (**Figures 6A, B** **)**. APase activity in the shoot of *atpap17* mutant line was higher than that of *atpap26* and *atpap17/atpap26* lines under +Pi conditions (**Figure 6A**), while no significant differences in root APase activity of mutant lines was observed under same conditions (**Figure 6B**). Also, our result revealed that APase activity in the shoots and roots of *atpap17* mutant line was significantly increased as compared to Col-0 under Pi-deficient conditions (**Figure 6**).

**Figure 7:**
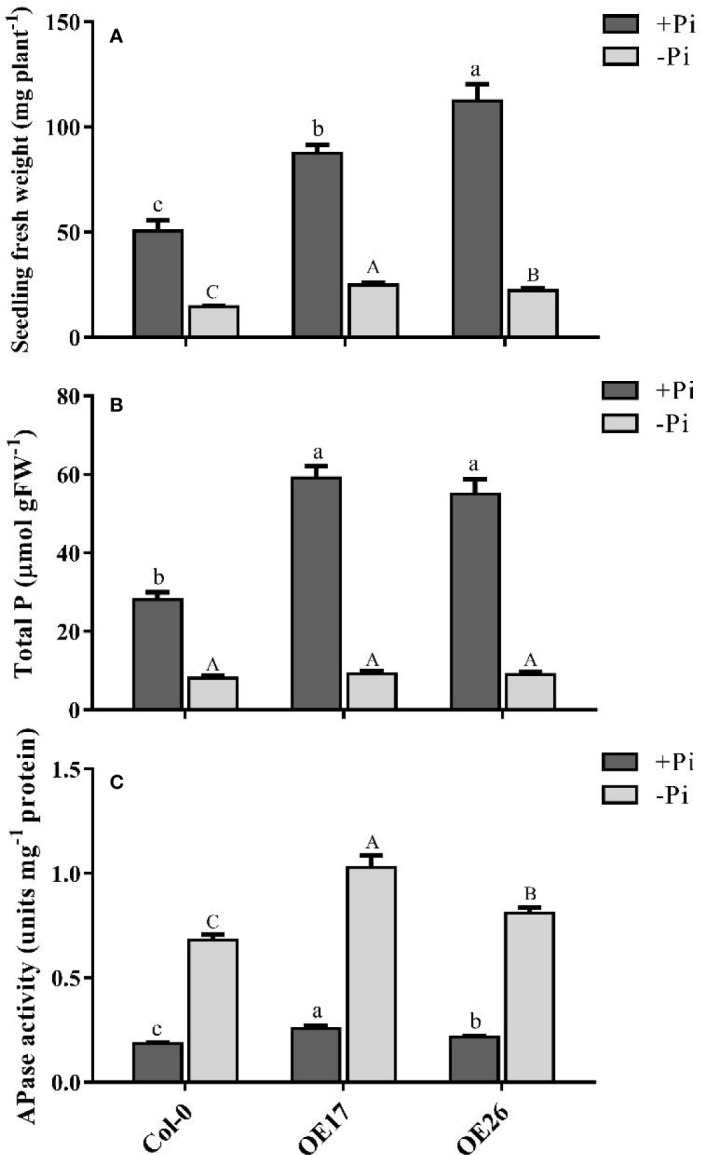
Analyses of *AtPAP17* and *AtPAP26* overexpressed lines (OE17 and OE26) grown under +Pi (1.25 mM KH_2_PO_4_) and −Pi (no Pi) conditions. Fresh weight **(A)**, total P content **(B)**, and APase activities **(C)** of 21-day-old Arabidopsis seedlings of Col-0, OE17 and OE26 lines. Each overexpressed line includes four independent transgenic lines. Values are the means ± SE of three biological replicates. Significant differences are indicated by different letters above the bars (*P* < 0.05).

For further functional analysis of *AtPAP17* and *AtPAP26* genes, the characteristics of *AtPAP17* and *AtPAP26* overexpressing lines, OE17 and OE26, were investigated under Pi-sufficient and Pi-deficient conditions. For this purpose, full length coding sequences of *AtPAP17* and *AtPAP26* genes under CaMV 35S promoter were introduced into WT plants (**Supplementary Figure S1**). The abundances of *AtPAP17* and *AtPAP26* transcripts in OE17 and OE26 plants, respectively, were higher than those of WT plants in both Pi conditions (**Figures 2E, F**, and **Supplementary Figure S1**). As shown in **Figure 7A**, FW of OE17 and OE26 lines was 1.7- and 2.2-folds, higher than that of Col-0 under Pi-sufficient conditions respectively. As expected, P content of OE17 and OE26 lines were higher than that in Col-0 (**Figure 7B**). These results were paralleled by APase activity increase in these lines (**Figure 7C** **)**. Investigating OE lines under Pi starvation showed that the FW of OE17 and OE26 were significantly higher than that of Col-0 (**Figure 7A** **)**. Generally, these results indicate that overexpression of *AtPAP17* and *AtPAP26* genes led to plant growth improvement under +Pi and −Pi conditions.

### Influence of *AtPAP17* and *AtPAP26* Genes on Root Structure

**Figure 8:**
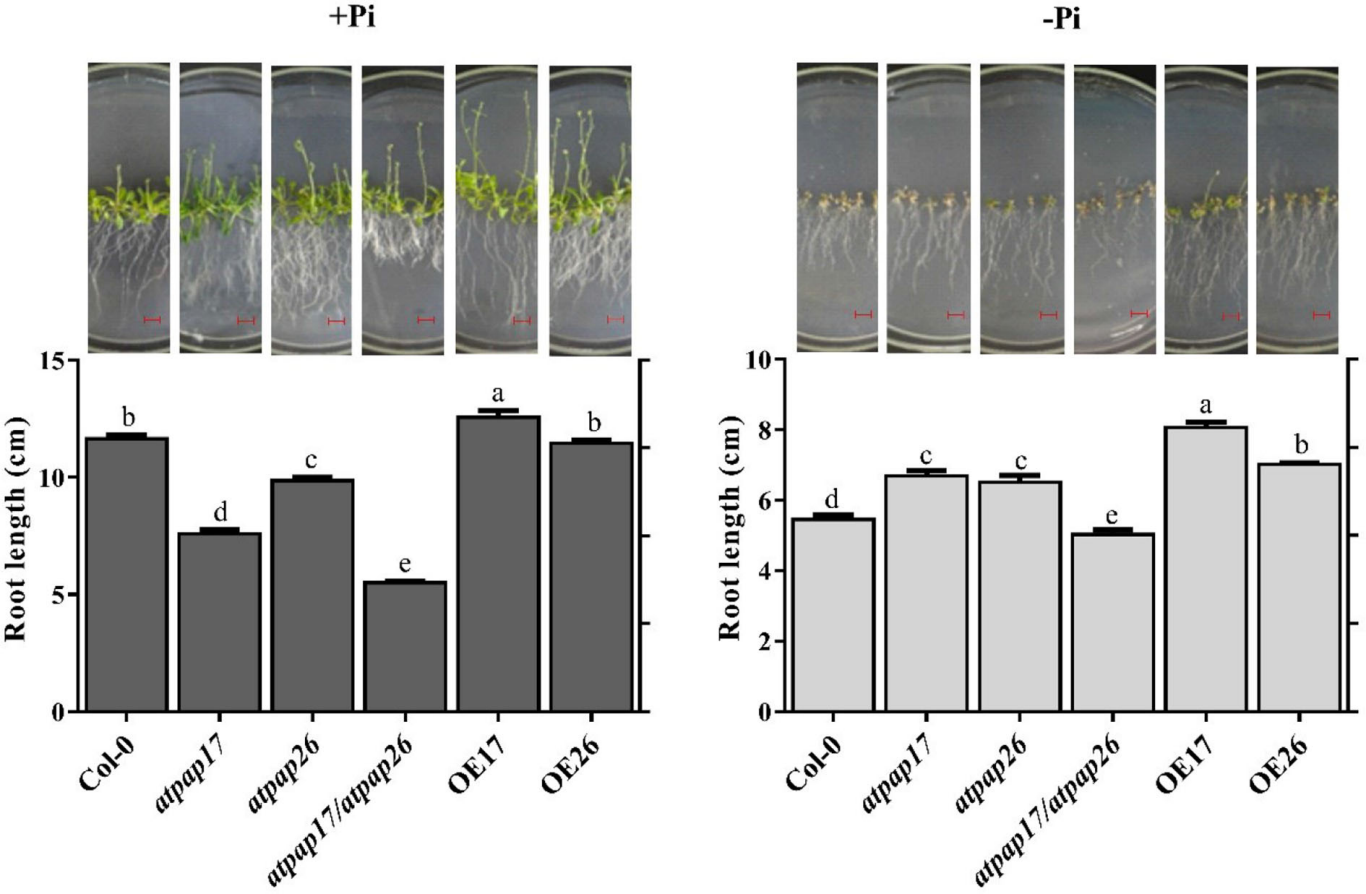
Main root length and morphology of Col-0, *AtPAP17* and *AtPAP26* mutants (*atpap17*, *atpap26*, and *atpap17/atpap26*) and overexpressing lines (OE17 and OE26) (each overexpressed line includes four independent transgenic lines) grown under +Pi (1.25 mM KH_2_PO_4_) and −Pi (no Pi) conditions. All lines were grown for 21 days on vertically oriented MS agar plates as described in the *Materials and Methods*. The pictures of plants grown under +Pi and −Pi are shown at the above columns. Values are the means ± SE of three biological replicates and each replicate is the mean of 12 seedlings. Significant differences are indicated by different letters above the bars (*P* < 0.05). Scale bars = 2 cm.

**Figure 9:**
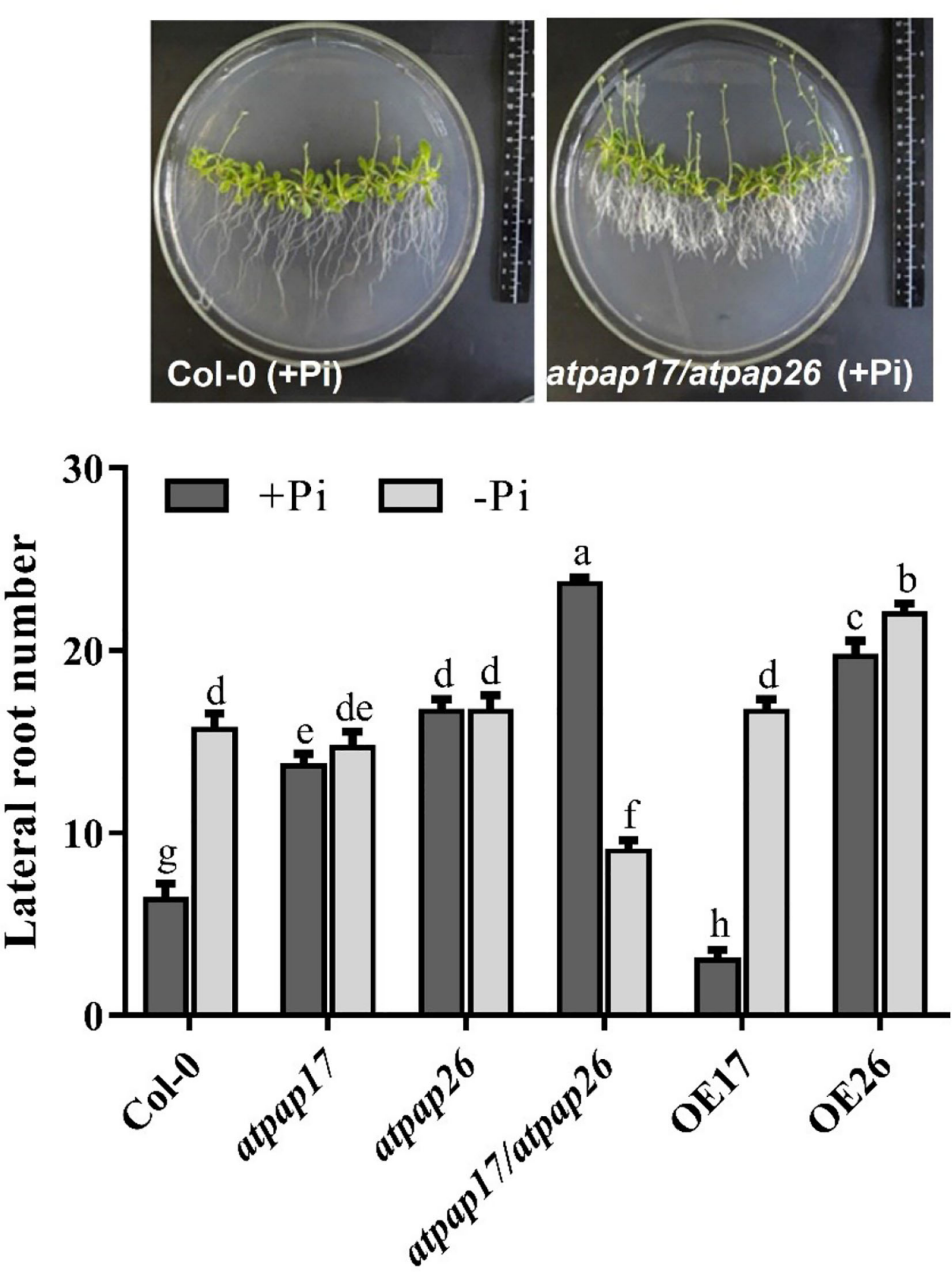
Lateral roots number of Col-0, *AtPAP17* and *AtPAP26* mutants (*atpap17*, *atpap26*, and *atpap17/atpap26*) and overexpressing lines (OE17 and OE26) (each overexpressed line includes four independent transgenic lines) grown under +Pi (1.25 mM KH_2_PO_4_) and −Pi (no Pi) conditions. All lines were grown for 21 days on vertically oriented MS agar plates as described in the *Materials and Methods*. Values are the means ± SE of three biological replicates and each replicate is the mean of 12 seedlings. Significant differences are indicated by different letters above the bars (*P* < 0.05).

Remodeling root system architecture including the inhibition of main root growth and formation of lateral and hairy roots is the strategy to cope with nutrient limitations such as Pi starvation (Ma et al., 2001; Williamson et al., 2001; Svistoonoff et al., 2007; Péret et al., 2011; Péret et al., 2014). As shown in **Figures 8**, **9**, the root architecture of plants was modified under different Pi conditions. The length of main roots in the plants under −Pi conditions was shorter than that under +Pi one. The destruction of *AtPAP17* and *AtPAP26* genes led to alternations in root architecture of mutant lines similar to that in Pi starvation (**Figure 8**). The length of main roots in *atpap17*, *atpap26*, and *atpap17/atpap26* mutant lines were 34, 15, and 52%, respectively, shorter than that in Col-0 under +Pi conditions (**Figure 8**). The shortest root length was related to *atpap17/atpap26* double mutant line under both +Pi and −Pi conditions. The root length of *atpap17/atpap26* double mutant line was 27 and 44% shorter than that of *atpap17* and *atpap26* single mutant lines, respectively, under +Pi conditions (**Figure 8**). This was associated with the formation of lateral and hairy roots in *atpap17/atpap26* double mutant line (**Figure 9**). The results showed that the number of lateral roots of *atpap17/*atpap26 double mutant line was 3.73-, 1.77-, and 1.42- folds higher than that of Col-0, *atpap17* and *atpap26* mutant lines, respectively, under +Pi conditions (**Figure 9**). Also, the lateral roots number of *atpap17* and *atpap26* single mutant lines were 2.15- and 2.63- folds higher than that of Col-0, respectively, under same conditions (**Figure 9**).

Root length increment of OE lines under Pi-sufficient and Pi-deficient conditions verified the influences of these two genes in Arabidopsis root structure. The highest root length was obtained for OE17 line in both +Pi and −Pi conditions (**Figure 8**). Results indicated that the root length of OE17 was 11% higher than that of Col-0 under Pi-sufficient conditions, while no significant differences was observed between root length of OE26 and Col-0 plants in the mentioned conditions (**Figure 8**). In comparison, OE17 and OE26 lines displayed root length increment by about 47 and 28.6%, respectively, higher than Col-0 under –Pi conditions. Also, the lowest number of lateral roots were found in OE17 line under +Pi conditions (**Figure 9**) while OE26 lateral roots number increased 3.10- and 1.40-folds as compared with Col-0 under +Pi and −Pi conditions, respectively. No significant differences were observed in lateral root numbers of *atpap17* and *atpap26* lines under −Pi conditions (**Figure 9**). All in all, *AtPAP17* and *AtPAP26* genes have an effective role in the morphology and development of Arabidopsis root system under different Pi conditions.

## Discussion

Phosphorus is a key element in cellular processes and its homeostasis is vital for plant growth and development (Ticconi and Abel, 2004; Chiou et al., 2006; Tran et al., 2010a). As a class of plant APases, several PAPs play important roles in plant adaptive responses to Pi limitation and its homeostasis maintenance. The analysis of microarray data showed that some PAPs markedly induced by Pi starvation (**Figure 1**). These ones, including *AtPAP14*, *AtPAP17*, and *AtPAP24* genes, were strongly induced in Arabidopsis seedlings during Pi starvation while returned to basal levels after Pi re-fed. Among them *AtPAP17* showed that the highest gene expression induction in response to Pi deficiency in root. This is consistent with previous studies (Del Pozo et al., 1999; Li et al., 2002). Therefore, *AtPAP17* gene, as a key phosphatase enzyme in response to Pi deficiency, was studied by reverse genetics approaches under different Pi conditions. In this study, a series of loss and gain function experiments were arranged in Pi sufficiency or deficiency conditions to elucidate the contributions of *AtPAP17* and *AtPAP26* and interaction between them in keeping Pi concentration within a certain range.

Unexpectedly, the biomass of *atpap17* mutant plants increased as compared to Col-0 under +Pi conditions (**Figure 3**). This was concurrent with a significant increase in P content of *atpap17* mutant seedlings (**Figure 4**). Given that *AtPAP17* gene is a member of biological network regulating Pi homeostasis in plant cells (Muller et al., 2007; Rouached et al., 2010), the increase of biomass and P content in *atpap17* mutant under Pi-sufficient conditions can be associated with the increment of the expression and activity of other APases. Therefore, the expression levels of some genes related to Pi homeostasis were investigated under Pi-sufficient conditions.

In this study, clustering based on transcription patterns of APases in response to various Pi conditions (data not shown), genes located in the same cluster with *AtPAP17* gene including *AtPAP26*, *AtPAP8*, *AtPLP3*, *AtHRP3*, *AtHRP9* were selected for further studies. The results showed that *AtPAP17* gene knockout resulted in a significant up-regulation of *HRP9*, *AtPAP8*, and *AtPAP26* genes in the seedling of *atpap17* single mutant (**Figure 5A**). Among these, *AtPAP26* gene was selected for two reasons: i) the expression level of this gene was relatively persistent regardless of Pi concentration in both shoot and root of Col-0 plant (**Figure 1**) while it was up-regulated in response to *AtPAP17* gene destruction (**Figure 5**), ii) *AtPAP17* has the highest expression change in root (**Figure 1B**) where *AtPAP26* gene expression was increased in *atpap17* mutant. The lack of phenotypic change or any detectable developmental defects in single mutants have been also observed by other researchers (Bouché and Bouchez, 2001; White et al., 2013; Gao et al., 2015; Kok et al., 2015).

Several mechanisms including genetic compensation have been proposed to explain such observations (Rossi et al., 2015; El-Brolosy and Stainier, 2017). Here, the presented data shows that there must be indirect connection among APase genes *via* sensing intracellular Pi concentration leading to compensatory regulation of others upon defect on a certain gene. One major strategy for functional compensation is transcriptional regulation as a vital biological process causes the cell and even whole organism respond to a range of extra- and intra-cellular signals. All of these regulations lead to keep homeostasis of cellular Pi needed for running metabolic reactions and so on.

In order to re-examine this hypothesis, *AtPAP26* T-DNA insertion mutant plants were inspected. DW and P content of *atpap26* line as well as *atpap17* line unexpectedly increased under +Pi conditions (**Figures 3B**, **4**). These results are consistent with the unanticipated increase in DW and P contents of 21-day-old soil-grown *atpap26* mutant as compared to Col-0 (Hurley et al., 2010). Our results showed that *AtPAP26* gene knockout was accompanied by *AtPAP17* expression increment in *atpap26* mutant under +Pi conditions. However, the result of this research and previous studies (Del Pozo et al., 1999; Veljanovski et al., 2006) indicated that *AtPAP17* gene has a very low expression in Col-0 plants under +Pi conditions.

Based on the unexpected results, (i) the increase of DW and P content in *atpap17* and *atpap26* single mutant lines as compared to Col-0, and (ii) the up-regulation of *AtPAP17* and *AtPAP26* genes in *atpap26* and *atpap17* single mutants, respectively, we extended our hypothesis to functional compensation through transcriptional adaptation as a mechanism to improve genomic competence against the loss of gene(s) function as pointed out previously in only few systems (Tondeleir et al., 2012; Rossi et al., 2015; Sztal et al., 2018; El-Brolosy et al., 2019). In this mechanism, the loss of function of one gene leads to the up-regulation of relevant genes that can undertake the function of the mutated gene (El-Brolosy and Stainier, 2017; Sztal et al., 2018; El-Brolosy et al., 2019).

To determine whether the compensatory up-regulation of *AtPAP17* and *AtPAP26* genes was accountable for the unexpected phenotype in *atpap17* and *atpap26* single mutant lines, the simultaneous knockout of *AtPAP17* and *AtPAP26* genes using T-DNA insertion were created by crossing *atpap17* line (pollen donor) with *atpap26* line (pollen receptor). The simultaneous knockout of these two genes significantly reduced DW and P content in *atpap17/atpap26* double mutant line as compare with single mutant lines. These data suggest that the double mutation is beyond genomic capacity for compensation of required APase activity. In other words, the contribution of other APase-encoding genes could not compensate for the simultaneous knockout of *AtPAP17* and *AtPAP26* genes in double mutant line.

Another major founding was that DW of *atpap17* line was more than that of other mutant lines under −Pi conditions. The higher DW in *atpap17* line as compared to other mutant lines (**Figure 3B**) can be attributed to dual role of the main component of compensatory network, *AtPAP26* gene (Hurley et al., 2010), in supplying Pi from internal and external P sources. Indeed, internal role of *AtPAP26* gene in mobilizing Pi from internal sources result in DW increment of *atpap17* line under −Pi conditions. Given that the compensatory function of *AtPAP17* gene in *atpap26* line under +Pi conditions was more effective than that under −Pi conditions (**Figure 4**), it seems that the corresponding enzyme probably contributed to acquire Pi from external sources (Del Pozo et al., 1999). Supplying Pi from external sources is accompanied by the activation of water transporters at root and water uptake. Therefore, the higher FW of *atpap26* line as compared to other examined mutant lines under +Pi conditions confirms the role of compensatory gene, *AtPAP17*, in supplying Pi from external sources. In this context, further studies on the expression of phosphate transporters under different Pi concentrations may help a better understanding.

The increase of DW, FW, and P content of *atpap26* line as compared to Col-0 under +Pi conditions was inconsistent with that had previously been reported in 14-day-old *atpap26* line in the same conditions (Wang et al., 2014). It is noteworthy that our analyses were carried out on 21-day-old seedlings. So, the activity of compensatory network followed by phosphate availability for an additional 7-day period likely resulted in the increment of DW and P content in *atpap26* line under +Pi condition in our study. These results are consistent with the increment of DW and P content in 21-day-old soil-grown *atpap26* line as compare with Col-0 under +Pi conditions (Hurley et al., 2010). In contrast, the longer period of Pi deficient stress might lead to a significant detrimental effect of *AtPAP26* gene knockout on the development of *atpap26* line under −Pi conditions. In other words, the activity of compensatory network in response to *AtPAP26* gene destruction could not fully compensate for *AtPAP26* gene knockout under −Pi condition due to the lack of P in culture medium. The adverse effects of *AtPAP26* gene knockout on −Pi *atpap26* line development were consistent with Hurley et al. (2010) study.

Under Pi deficient conditions, the expression of several PAP-encoding genes was activated (**Figures 1**, **5**), thus phosphatase activity of −Pi plants is higher than that under +Pi conditions. The reduction of APase activity in mutant plants as compared to Col-0 plants was inconsistent with the increased expression levels of *HRP9*, *AtPAP8*, and *AtPAP26* genes in *atpap17* line and *AtPAP17*, *AtPAP8*, and *HRP3* genes in *atpap26* line. Apparently, the APase activity depends on the nature of enzyme encoded by the activated gene(s) in response to the loss of gene(s) function, and/or the type of substrate in this enzyme assay. For example, phosphoenolpyruvate (PEP) was known as the preferred substrate for AtPAP26 enzyme (Veljanovski et al., 2006). Thus, measured APase activity reduction in the mutant plants can be due to the non-specific substrate used in this study, *p*NPP. It is also reported that APase activity using *p*NPP substrate in *atpap12/atpap26* double mutant line was 46 and 55% lower than that in control plant under +Pi and −Pi conditions, respectively (Robinson et al., 2012b). Meanwhile, the specific substrate of AtPAP17 enzyme is unknown.

Our results showed *AtPAP17* gene destruction resulted in APase increment in *atpap17* seedlings under −Pi conditions. Also, no significant difference was detected between *atpap26* line and Col-0 in root APase activity under same conditions. These results are inconsistent with the previous studies (Tran et al., 2010b; Wang et al., 2014). This may be due to different growth period as well as various Pi deficient term. So that in our study, APase activity analysis was performed on 21-day-old seedlings while in previous studies (Tran et al., 2010b; Wang et al., 2014), 14-day-old seedlings were analyzed for APase activity measurement. Indeed, in this study the seedlings were exposed to an additional 7-day period of Pi deficient stress as compared to previous studies (Tran et al., 2010b; Wang et al., 2014). Therefore, the longer period of Pi deficient stress likely resulted in the increase of APase activity following the induction of more APase genes in *atpap17* and *atpap26* single mutant lines. It has been documented that some genes involved in Pi mobilization from organic compounds were induced upon prolonged Pi deficiency (Misson et al., 2005). Li et al. (2017) has also been reported that *GmPAP21* gene expression was highly induced by long term Pi deficiency in soybean roots. It is noteworthy that APase activity of *atpap17/atpap26* double mutant line under −Pi conditions was lower than that of Col-0, probably indicating the high share of *AtPAP17* and *AtPAP26* genes in APase activity under −Pi conditions.

The nutrient availability in various environmental conditions is associated with alterations in root architecture (Malamy, 2005; Ristova and Busch, 2014). The results showed that plant main root length under -Pi conditions was reduced as compared to that under +Pi conditions. Main root longitudinal growth reduction and the increase of lateral roots number are general plant responses to the nutritional stresses (Péret et al., 2011). Interestingly, the structure alteration of *atpap17/atpap26* double mutant roots (lateral root formation and severe decrease in main root length) were observed under +Pi conditions, despite Pi presence in culture medium. Indeed, root structure changes in *atpap17/atpap26* line grown on Pi-sufficient conditions were similar to root structural response to Pi starvation (**Figure 8**). It seems that the simultaneous knockout of *AtPAP17* and *AtPAP26* genes significantly affected root structure due to disturbance in APase activity and thus P content even under +Pi conditions. Overall, root structural alterations can be explained by two hypotheses: i) a nutritional response; and ii) a signaling response (Svistoonoff et al., 2007). P content decrement of *atpap17/atpap26* double mutant line under +Pi conditions is supported by nutritional response hypothesis. In fact, simultaneous knockout of *AtPAP17* and *AtPAP26* enzymes reduced the ability of *atpap17/atpap26* double mutant line to supply P, despite Pi presence in culture medium, and thus the root morphology changed to compensate for Pi deficiency. According to signaling response hypothesis, environmental conditions sensed by root tip, regardless of internal P content in cells determines primary root growth pattern and also root architecture changes (Svistoonoff et al., 2007). Root structure of the plants grown under −Pi conditions is supported by signaling response hypothesis. While root structural alterations in *atpap17/atpap26* double mutant line grown under +Pi conditions and Pi presence in culture medium, cannot be explained by this hypothesis. Studying Low Pi Root (LPR) genes in root tip and genes involved in phytohormone responses including auxin (Svistoonoff et al., 2007; Pérez-Torres et al., 2008) in *atpap17/atpap26* double mutant line can provide a more accurate understanding of root structural changes in this plant under +Pi conditions.

The lower growth of main root in *atpap17* line, as compared to *atpap26* line, indicate the higher effect of *AtPAP17* gene on root growth directly or indirectly. Increase root length of OE17 plants as compared to other plants also support this idea. In our current study, heterologous expression of *AtPAP17* gene in tobacco resulted in higher root longitudinal growth of transgenic plants (data not shown). Consistently, the root length of OE26 plants displayed no significant difference with Col-0 under +Pi conditions. Meanwhile DW of OE26 roots was higher than that of Col-0 under different Pi concentrations which was due to the high number of lateral roots (**Supplementary Figure S2**). This allows exploration of wide area for Pi scavenging in soil result in high accumulation of Pi in OE26 line (**Figure 7B**). According to these results, it can be concluded that *AtPAP17* and *AtPAP26* genes affect plant root structure and expression of these genes help plants with nutrient uptake. Also, OEs growth improvement (**Figure 7**) suggested that these genes increase plant Pi use efficiency, and can potentially be used for crop improvement.

In summary, here we have uncovered a novel case of genetic compensation within PAPs gene family of Arabidopsis plant for the first time. Our results showed that there is a compensation relationship between *AtPAP17* and *AtPAP26* genes in Pi homeostasis. Furthermore, the data reported here indicated that the study of interactions between genes provides a better insight in biological processes regulation in the cell. Since *AtPAP17* and *AtPAP26* genes are only known PAPs involved in Pi remobilization from senescing leaves to younger sink tissue (Robinson et al., 2012a; Stigter and Plaxton, 2015), it will be of interest to assess the effects of *atpap17/atpap26* double mutant and also *AtPAP17* and *AtPAP26* overexpressed lines on Pi metabolism, APase activities, *etc*., during leaf senescence as well.

## Data Availability Statement

All datasets presented in this study are included in the article/**Supplementary Material**.

## Author Contributions

SF carried out all experiments and analyses, and wrote the manuscript. MS directed the research. MM and AM advised the experiments. All authors contributed to the article and approved the submitted version.

## Conflict of Interest

The authors declare that the research was conducted in the absence of any commercial or financial relationships that could be construed as a potential conflict of interest.

## References

[B1] Alonso J. M. Stepanova A. N. Leisse T. J. Kim C. J. Chen H. Shinn P. (2003). Genome-Wide Insertional Mutagenesis of *Arabidopsis thaliana* . Science 301 (5633), 653. 10.1126/science.1086391 12893945

[B2] Ames B. N. (1966). “Assay of inorganic phosphate, total phosphate and phosphatases,” in Methods in Enzymology. Eds. Neufeld E. F. Ginsburg V. (Cambridge, FL: Academic Press), 115–118.

[B3] Bhadouria J. Singh A. P. Mehra P. Verma L. Srivastawa R. Parida S. K. (2017). Identification of Purple Acid Phosphatases in Chickpea and Potential Roles of CaPAP7 in Seed Phytate Accumulation. Sci. Rep. 7 (1), 11012. 10.1038/s41598-017-11490-9 28887557 PMC5591292

[B4] Bouché N. Bouchez D. (2001). Arabidopsis gene knockout: phenotypes wanted. Curr. Opin. Plant Biol. 4 (2), 111–117. 10.1016/S1369-5266(00)00145-X 11228432

[B5] Bradford M. M. (1976). A rapid and sensitive method for the quantitation of microgram quantities of protein utilizing the principle of protein-dye binding. Anal. Biochem. 72, 248–254. 10.1016/0003-2697(76)90527-3 942051

[B6] Chiou T.-J. Aung K. Lin S.-I. Wu C.-C. Chiang S.-F. Su C.-L. (2006). Regulation of Phosphate Homeostasis by MicroRNA in *Arabidopsis thaliana* . Plant Cell 18 (2), 412. 10.1105/tpc.105.038943 16387831 PMC1356548

[B7] Del Pozo J. C. Allona I. Rubio V. Leyva A. De La Peña A. Aragoncillo C. (1999). A type 5 acid phosphatase gene from Arabidopsis thaliana is induced by phosphate starvation and by some other types of phosphate mobilising/oxidative stress conditions. Plant J. 19 (5), 579–589. 10.1046/j.1365-313X.1999.00562.x 10504579

[B8] Duff S. M. Sarath G. Plaxton W. C. (1994). The role of acid phosphatases in plant phosphorus metabolism. Physiol. Plant. 90 (4), 791–800. 10.1111/j.1399-3054.1994.tb02539.x

[B9] El-Brolosy M. A. Stainier D. Y. (2017). Genetic compensation: A phenomenon in search of mechanisms. PloS Genet. 13 (7), e1006780. 10.1371/journal.pgen.1006780 28704371 PMC5509088

[B10] El-Brolosy M. A. Kontarakis Z. Rossi A. Kuenne C. Guenther S. Fukuda N. (2019). Genetic compensation triggered by mutant mRNA degradation. Nature 568, 193–197. 10.1038/s41586-019-1064-z 30944477 PMC6707827

[B11] Fang Z. Shao C. Meng Y. Wu P. Chen M. (2009). Phosphate signaling in Arabidopsis and Oryza sativa. Plant Sci. 176 (2), 170–180. 10.1016/j.plantsci.2008.09.007

[B12] Gao Y. Zhang Y. Zhang D. Dai X. Estelle M. Zhao Y. (2015). Auxin binding protein 1 (ABP1) is not required for either auxin signaling or Arabidopsis development. Proc. Natl. Acad. Sci. 112 (7), 2275–2280. 10.1073/pnas.1500365112 25646447 PMC4343106

[B13] Hammond J. P. Broadley M. R. White P. J. (2004). Genetic responses to phosphorus deficiency. Ann. Bot. 94 (3), 323–332. 10.1093/aob/mch156 15292042 PMC4242181

[B14] Haran S. Logendra S. Seskar M. Bratanova M. Raskin I. (2000). Characterization of Arabidopsis Acid Phosphatase Promoter and Regulation of Acid Phosphatase Expression. Plant Physiol. 124 (2), 615. 10.1104/pp.124.2.615 11027712 PMC59168

[B15] Huang C. Y. Roessner U. Eickmeier I. Genc Y. Callahan D. L. Shirley N. (2008). Metabolite profiling reveals distinct changes in carbon and nitrogen metabolism in phosphate-deficient barley plants (Hordeum vulgare L.). Plant Cell Physiol. 49 (5), 691–703. 10.1093/pcp/pcn044 18344526

[B16] Hurley B. A. Tran H. T. Marty N. J. Park J. Snedden W. A. Mullen R. T. (2010). The dual-targeted purple acid phosphatase isozyme AtPAP26 is essential for efficient acclimation of Arabidopsis to nutritional phosphate deprivation. Plant Physiol. 153 (3), 1112–1122. 10.1104/pp.110.153270 20348213 PMC2899917

[B17] Kok F. O. Shin M. Ni C.-W. Gupta A. Grosse A. S. van Impel A. (2015). Reverse genetic screening reveals poor correlation between morpholino-induced and mutant phenotypes in zebrafish. Dev. Cell 32 (1), 97–108. 10.1016/j.devcel.2014.11.018 25533206 PMC4487878

[B18] Kong Y. Li X. Wang B. Li W. Du H. Zhang C. (2018). The Soybean Purple Acid Phosphatase GmPAP14 Predominantly Enhances External Phytate Utilization in Plants. Front. Plant Sci. 9, 292. 10.3389/fpls.2018.00292 29593758 PMC5857590

[B19] Kuang R. Chan K.-H. Yeung E. Lim B. L. (2009). Molecular and biochemical characterization of AtPAP15, a purple acid phosphatase with phytase activity, in Arabidopsis. Plant Physiol. 151 (1), 199–209. 10.1104/pp.109.143180 19633233 PMC2735976

[B20] Li D. Zhu H. Liu K. Liu X. Leggewie G. Udvardi M. (2002). Purple acid phosphatases of Arabidopsis thaliana comparative analysis and differential regulation by phosphate deprivation. J. Biol. Chem. 277 (31), 27772–27781. 10.1074/jbc.M204183200 12021284

[B21] Li C. Li C. Zhang H. Liao H. Wang X. (2017). The purple acid phosphatase *GmPAP21* enhances internal phosphorus utilization and possibly plays a role in symbiosis with rhizobia in soybean. Physiol. Plant. 159 (2), 215–227. 10.1111/ppl.12524 27762446

[B22] Liang C. Wang J. Zhao J. Tian J. Liao H. (2014). Control of phosphate homeostasis through gene regulation in crops. Curr. Opin. Plant Biol. 21, 59–66. 10.1016/j.pbi.2014.06.009 25036899

[B23] Liu P.-D. Xue Y.-B. Chen Z.-J. Liu G.-D. Tian J. (2016). Characterization of purple acid phosphatases involved in extracellular dNTP utilization in Stylosanthes. J. Exp. Bot. 67 (14), 4141–4154. 10.1093/jxb/erw190 27194738 PMC5301924

[B24] Livak K. J. Schmittgen T. D. (2001). Analysis of relative gene expression data using real-time quantitative PCR and the 2^– ΔΔCT^ method. Methods 25 (4), 402–408. 10.1006/meth.2001.1262 11846609

[B25] Lohrasebi T. Malboobi M. A. Samaeian A. Sanei V. (2007). Differential expression of Arabidopsis thaliana acid phosphatases in response to abiotic stresses. Iran. J. Biotechnol. 5 (3), 130–139.

[B26] López-Arredondo D. L. Leyva-González M. A. González-Morales S. I. López-Bucio J. Herrera-Estrella L. (2014). Phosphate nutrition: improving low-phosphate tolerance in crops. Annu. Rev. Plant Biol. 65, 95–123. 10.1146/annurev-arplant-050213-035949 24579991

[B27] Ma Z. Bielenberg D. Brown K. Lynch J. (2001). Regulation of root hair density by phosphorus availability in Arabidopsis thaliana. Plant Cell Environ. 24 (4), 459–467. 10.1046/j.1365-3040.2001.00695.x

[B28] Malamy J. (2005). Intrinsic and environmental response pathways that regulate root system architecture. Plant Cell Environ. 28 (1), 67–77. 10.1111/j.1365-3040.2005.01306.x 16021787

[B29] Misson J. Raghothama K. G. Jain A. Jouhet J. Block M. A. Bligny R. (2005). A genome-wide transcriptional analysis using Arabidopsis thaliana Affymetrix gene chips determined plant responses to phosphate deprivation. Proc. Natl. Acad. Sci. 102 (33), 11934–11939. 10.1073/pnas.0505266102 16085708 PMC1188001

[B30] Muller R. Morant M. Jarmer H. Nilsson L. Hamborg Nielsen T. H. (2007). Genome-wide analysis of the Arabidopsis leaf transcriptome reveals interaction of phosphate and sugar metabolism. Plant Physiol. 143, 156–171. 10.1104/pp.106.090167 17085508 PMC1761981

[B31] Murashige T. Skoog F. (1962). A revised medium for rapid growth and bio assays with tobacco tissue cultures. Physiol. Plant. 15 (3), 473–497. 10.1111/j.1399-3054.1962.tb08052.x

[B32] Naseri J. I. Truong N. T. Hörentrup J. Kuballa P. Vogel A. Rompel A. (2004). Porcine purple acid phosphatase: heterologous expression, characterization, and proteolytic analysis. Arch. Biochem. Biophys. 432 (1), 25–36. 10.1016/j.abb.2004.08.008 15519293

[B33] Olczak M. Morawiecka B. Watorek W. (2003). Plant purple acid phosphatases - genes, structures and biological function. Acta Biochim. Pol. 50 (4), 1245–1256. 10.18388/abp.2003_3648 14740011

[B34] Péret B. Clément M. Nussaume L. Desnos T. (2011). Root developmental adaptation to phosphate starvation: better safe than sorry. Trends Plant Sci. 16 (8), 442–450. 10.1016/j.tplants.2011.05.006 21684794

[B35] Péret B. Desnos T. Jost R. Kanno S. Berkowitz O. Nussaume L. (2014). Root Architecture Responses: In Search of Phosphate. Plant Physiol. 166 (4), 1713. 10.1104/pp.114.244541 25341534 PMC4256877

[B36] Pérez-Torres C.-A. López-Bucio J. Cruz-Ramírez A. Ibarra-Laclette E. Dharmasiri S. Estelle M. (2008). Phosphate availability alters lateral root development in Arabidopsis by modulating auxin sensitivity via a mechanism involving the TIR1 auxin receptor. Plant Cell 20 (12), 3258–3272. 10.1105/tpc.108.058719 19106375 PMC2630440

[B37] Plaxton W. C. Tran H. T. (2011). Metabolic adaptations of phosphate-starved plants. Plant Physiol. 156 (3), 1006–1015. 10.1104/pp.111.175281 21562330 PMC3135920

[B38] Poirier Y. Bucher M. (2002). Phosphate transport and homeostasis in Arabidopsis. The Arabidopsis book, 1, e0024. 10.1199/tab.0024 22303200 PMC3243343

[B39] Raghothama K. (1999). Phosphate acquisition. Annu. Rev. Plant Biol. 50 (1), 665–693. 10.1146/annurev.arplant.50.1.665 15012223

[B40] Richardson A. E. (2009). Regulating the phosphorus nutrition of plants: molecular biology meeting agronomic needs. Plant Soil 322 (1-2), 17–24. 10.1007/s11104-009-0071-5

[B41] Ristova D. Busch W. (2014). Natural variation of root traits: from development to nutrient uptake. Plant Physiol. 166 (2), 518–527. 10.1104/pp.114.244749 25104725 PMC4213084

[B42] Robinson W. D. Carson I. Ying S. Ellis K. Plaxton W. C. (2012a). Eliminating the purple acid phosphatase AtPAP26 in Arabidopsis thaliana delays leaf senescence and impairs phosphorus remobilization. New Phytol. 196 (4), 1024–1029. 10.1111/nph.12006 23072540

[B43] Robinson W. D. Park J. Tran H. T. Del Vecchio H. A. Ying S. Zins J. L. (2012b). The secreted purple acid phosphatase isozymes AtPAP12 and AtPAP26 play a pivotal role in extracellular phosphate-scavenging by Arabidopsis thaliana. J. Exp. Bot. 63 (18), 6531–6542. 10.1093/jxb/ers309 23125358 PMC3504502

[B44] Rossi A. Kontarakis Z. Gerri C. Nolte H. Hölper S. Krüger M. (2015). Genetic compensation induced by deleterious mutations but not gene knockdowns. Nature 524 (7564), 230. 10.1038/nature14580 26168398

[B45] Rouached H. Arpat A. B. Poirier Y. (2010). Regulation of phosphate starvation responses in plants: signaling players and cross-talks. Mol. Plant 3 (2), 288–299. 10.1093/mp/ssp120 20142416

[B46] Sabet M. S. Zamani K. Lohrasebi T. Malboobi M. A. Valizadeh M. (2018). Functional Assessment of an Overexpressed Arabidopsis Purple Acid Phosphatase Gene (AtPAP26) in Tobacco Plants. Iran. J. Biotechnol. 16 (1), 31–41. 10.21859/IJB.2024 PMC6217264 30555844

[B47] Shen J. Yuan L. Zhang J. Li H. Bai Z. Chen X. (2011). Phosphorus dynamics: from soil to plant. Plant Physiol. 156 (3), 997–1005. 10.1104/pp.111.175232 21571668 PMC3135930

[B48] Song L. Liu D. (2015). Ethylene and plant responses to phosphate deficiency. Front. Plant Sci. 6, 796. 10.3389/fpls.2015.00796 26483813 PMC4586416

[B49] Stigter K. A. Plaxton W. C. (2015). Molecular mechanisms of phosphorus metabolism and transport during leaf senescence. Plants 4 (4), 773–798. 10.3390/plants4040773 27135351 PMC4844268

[B50] Stutter M. I. Shand C. A. George T. S. Blackwell M. S. Bol R. MacKay R. L. (2012). Recovering phosphorus from soil: a root solution? Environ. Sci. Technol. 46 (4), 1977–1978. 10.1021/es2044745 22280364

[B51] Svistoonoff S. Creff A. Reymond M. Sigoillot-Claude C. Ricaud L. Blanchet A. (2007). Root tip contact with low-phosphate media reprograms plant root architecture. Nat. Genet. 39 (6), 792. 10.1038/ng2041 17496893

[B52] Sztal T. E. McKaige E. A. Williams C. Ruparelia A. A. Bryson-Richardson R. J. (2018). Genetic compensation triggered by actin mutation prevents the muscle damage caused by loss of actin protein. PloS Genet. 14 (2), e1007212. 10.1371/journal.pgen.1007212 29420541 PMC5821405

[B53] Tian J. Liao H. (2015). “The role of intracellular and secreted purple acid phosphatases in plant phosphorus scavenging and recycling,” in Annual Plant Review: Phosphorus Metabolism in Plants. Eds. Plaxton W. C. Lambers H. (Hoboken, FL: Wiely), 265–288. 10.1002/9781118958841.ch10

[B54] Tian J. Wang C. Zhang Q. He X. Whelan J. Shou H. (2012). Overexpression of OsPAP10a, a root-associated acid phosphatase, increased extracellular organic phosphorus utilization in rice. J. Integr. Plant Biol. 54 (9), 631–639. 10.1111/j.1744-7909.2012.01143.x 22805094

[B55] Ticconi C. A. Abel S. (2004). Short on phosphate: plant surveillance and countermeasures. Trends Plant Sci. 9 (11), 548–555. 10.1016/j.tplants.2004.09.003 15501180

[B56] Tondeleir D. Lambrechts A. Müller M. Jonckheere V. Doll T. Vandamme D. (2012). Cells lacking β-actin are genetically reprogrammed and maintain conditional migratory capacity. Mol. Cell. Proteomics 11 (8), 255–271. 10.1074/mcp.M111.015099 22448045 PMC3412960

[B57] Tran H. T. Hurley B. A. Plaxton W. C. (2010a). Feeding hungry plants: the role of purple acid phosphatases in phosphate nutrition. Plant Sci. 179 (1-2), 14–27. 10.1016/j.plantsci.2010.04.005

[B58] Tran H. T. Qian W. Hurley B. A. SHE Y. M. Wang D. Plaxton W. C. (2010b). Biochemical and molecular characterization of AtPAP12 and AtPAP26: the predominant purple acid phosphatase isozymes secreted by phosphate-starved Arabidopsis thaliana. Plant Cell Environ. 33 (11), 1789–1803. 10.1111/j.1365-3040.2010.02184.x 20545876

[B59] Vance C. P. Uhde-Stone C. Allan D. L. (2003). Phosphorus acquisition and use: critical adaptations by plants for securing a nonrenewable resource. New Phytol. 157 (3), 423–447. 10.1046/j.1469-8137.2003.00695.x 33873400

[B60] Veljanovski V. Vanderbeld B. Knowles V. L. Snedden W. A. Plaxton W. C. (2006). Biochemical and molecular characterization of AtPAP26, a vacuolar purple acid phosphatase up-regulated in phosphate-deprived Arabidopsis suspension cells and seedlings. Plant Physiol. 142 (3), 1282–1293. 10.1104/pp.106.087171 16963519 PMC1630754

[B61] Wang L. Liu D. (2018). Functions and regulation of phosphate starvation-induced secreted acid phosphatases in higher plants. Plant Sci. 271, 108–116. 10.1016/j.plantsci.2018.03.013 29650148

[B62] Wang L. Li Z. Qian W. Guo W. Gao X. Huang L. (2011). The Arabidopsis purple acid phosphatase AtPAP10 is predominantly associated with the root surface and plays an important role in plant tolerance to phosphate limitation. Plant Physiol. 157 (3), 1283–1299. 10.1104/pp.111.183723 21941000 PMC3252131

[B63] Wang L. Lu S. Zhang Y. Li Z. Du X. Liu D. (2014). Comparative genetic analysis of Arabidopsis purple acid phosphatases AtPAP10, AtPAP12, and AtPAP26 provides new insights into their roles in plant adaptation to phosphate deprivation. J. Integr. Plant Biol. 56 (3), 299–314. 10.1111/jipb.12184 24528675

[B64] White J. K. Gerdin A.-K. Karp N. A. Ryder E. Buljan M. Bussell J. N. (2013). Genome-wide generation and systematic phenotyping of knockout mice reveals new roles for many genes. Cell 154 (2), 452–464. 10.1016/j.cell.2013.06.022 23870131 PMC3717207

[B65] Williamson L. C. Ribrioux S. P. Fitter A. H. Leyser H. O. (2001). Phosphate availability regulates root system architecture in Arabidopsis. Plant Physiol. 126 (2), 875–882. 10.1104/pp.126.2.875 11402214 PMC111176

[B66] Woo J. MacPherson C. R. Liu J. Wang H. Kiba T. Hannah M. A. (2012). The response and recovery of the Arabidopsis thaliana transcriptome to phosphate starvation. BMC Plant Biol. 12 (1), 62. 10.1186/1471-2229-12-62 22553952 PMC3520718

[B67] Yuan H. Liu D. (2008). Signaling components involved in plant responses to phosphate starvation. J. Integr. Plant Biol. 50 (7), 849–859. 10.1111/j.1744-7909.2008.00709.x 18713395

[B68] Zamani K. Sabet M. S. Lohrasebi T. Mousavi A. Malboobi M. A. (2012). Improved phosphate metabolism and biomass production by overexpression of AtPAP18 in tobacco. Biologia 67 (4), 713–720. 10.2478/s11756-012-0072-3

[B69] Zamani K. Lohrasebi T. Sabet M. S. Malboobi M. A. Mousavi A. (2014). Expression pattern and subcellular localization of Arabidopsis purple acid phosphatase AtPAP9. Gene Expression Patterns 14 (1), 9–18. 10.1016/j.gep.2013.08.001 24012521

[B70] Zhang Y. Wang X. Lu S. Liu D. (2014). A major root-associated acid phosphatase in Arabidopsis, AtPAP10, is regulated by both local and systemic signals under phosphate starvation. J. Exp. Bot. 65 (22), 6577–6588. 10.1093/jxb/eru377 25246445 PMC4246188

