# Supplementary material for: The Critical Role of AtPAP17 and AtPAP26 Genes in Arabidopsis Phosphate Compensation Network
Source: Front Plant Sci. 2020 Sep 30;11:565865. doi: 10.3389/fpls.2020.565865 (PMC7554520; doi:10.3389/fpls.2020.565865)
Supplement: Supplementary file 1 [file DataSheet_1.pdf]

Supplementary Information

The Critical Role of *AtPAP17* and *AtPAP26* Genes in Arabidopsis Phosphate Compensation Network

Siamak Farhadi[1], Mohammad Sadegh Sabet[1*], Mohammad Ali Malboobi[2] and Ahmad Moieni[1]

[1]Department of Plant Genetics and Breeding, Faculty of Agriculture, Tarbiat Modares University, Tehran, P.O. Box: 14115-336, Iran.

[2]Department of Plant Biotechnology, National Institute of Genetic Engineering and Biotechnology, Tehran, Iran.

*Corresponding author: ms.sabet@modares.ac.ir

Table S1. Sequences of the primers used for PCR-screening of mutants and expression analysis of APase genes

**Gene Name**	**Primer Name**	**Primer Sequence** (5'- 3')
AtPAP26	UniqPAP26-F	TAGGCGATATGGGTCAGACATTC
	UniqPAP26-R	CAGCGTACCAAAGAGGACTGCTAC
	PAP26-F	TCGAGGGATCCTGACGTGATGAATCATTTG
	APase5-R	ACTTGGATCCAAAGCATGAGGCAGACT
AtPAP17	AP29-F	CGAGTCTGAGTTTGCTGTTGT
	AP29-R	ACATAAGAGTTGCGAGATGGAAC
	PAP17m-F	CCACGGCGAGTCTGAGTTTGCTGTTGTGTA
	PAP17m-R	GGAGGCAGTTACTTTCGGGTCGTTCCTTTG
α-Tubulin	Tub-F	GCTTTCAACACCTTCTTCAG
	Tub-R	GAATAGTTCGCTTGGTCTT
AtHRP2	HRP2-F	GAACTTGGCTTCACTGATTTGT
	HRP2-R	AGCAGCTATGAGGGGATTTAGT
AtHRP3	HRP3-F	GAAGATATGGAGAGGATACTAATGG
	HRP3-R	AAGAAGTGATACGAAAAATGAGC
AtHRP9	HRP9-F	GCATATTTTTACGCCAAAGGACT
	HRP9-R	TCTCCAATATTCCCAACGATGT
AtPLP3	PLP3-F	GCCATGGTGATAAAAGCGTCATA
	PLP3-R	ACTCTCGCAGCCTCCAACACT
AtPAP2	UniPAP2-F	ACTAAAATCCCAAATCTGAAAAC
	UniPAP2-R	TTATGTCTCCTCGTTCTTGACTG
AtPAP8	UniPAP8-F	GCCTATCAAACTCATATTTTCTATTTTC
	UniPAP8-R	GTAATACGCCTCTCCAAT
AtPAP12	UniPAP12-F	CCAGATGACATGCCACTAGACAGCG
	UniPAP12-R	CGAACCTTCCCCAAGTATCCCAT
Actin	Actin-qPCR-F	GACCTTGCTGGACGTGACCTTAC
	Actin- qPCR-R	TAGTCAACAGCAACAAAGGAGAGC
AtPAP26	PAP26-qPCR-F	TAGGCGATATGGGTCAGACATTC
	PAP26-qPCR-R	CGGTACTACGCTCCACAAAACGA
AtPAP17	PAP17-qPCR-F	TTTGTCGTTGATGCAGAGTTGGTAG
	PAP17-qPCR-R	ACATAAGAGTTGCGAGATGGAAC
T-DNA	LBb1	GCGTGGACCGCTTGCTGCAACT

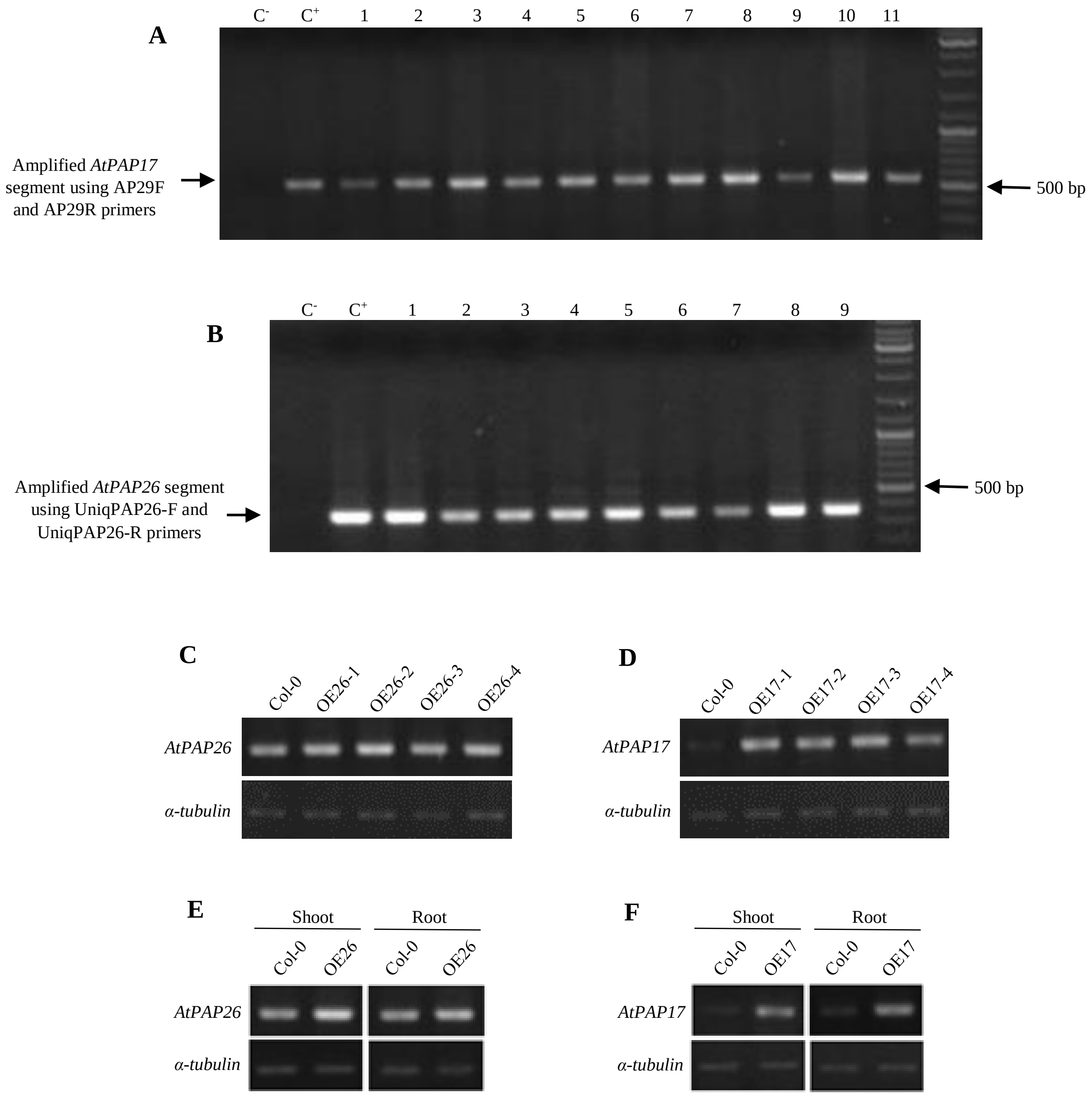

Fig. S1. Evaluation and verification of homozygous *AtPAP17* (**A**) and *AtPAP26* (**B**) overexpression lines (T_3 lines) by PCR of gDNA using *AtPAP17*- and *AtPAP26*-specific primers. Line 1-11 in **A** and 1-9 in **B** are independent homozygous transgenic lines, C^+: pARM2-AtPAP17 and pARM1-AtPAP26 constructs (Sabet *et al*., 2018) were used as positive control in **A** and **B**, respectively. C^-, all essential components of the amplification reaction except the template, used as a negative control. Semi-quantitative RT-PCR analysis of *AtPAP26* (**C**) and *AtPAP17* (**D**) transcript abundances in *AtPAP17* and *AtPAP26* overexpression lines used in this study using *AtPAP17*- and *AtPAP26*-specific primers. OE17-1 to OE17-4 are four independent OE17 lines, and also OE26-1 to OE26-4 are four independent OE26 lines. Semi-quantitative RT-PCR analysis of *AtPAP26* (**E**) and *AtPAP17* (**F**) in shoots and roots of OE26 and OE17 lines, respectively. Housekeeping gene *α-tubulin* was used as an internal control for normalization.

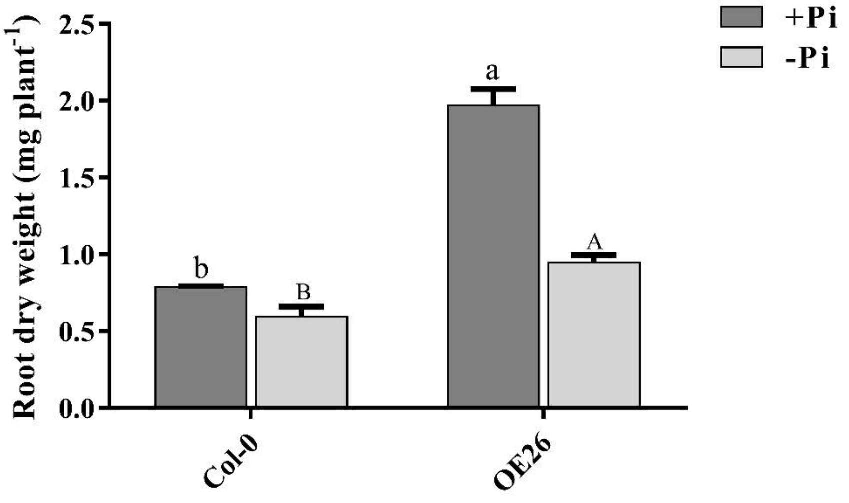

Fig. S2. Root dry weight of Col-0 and *AtPAP26*-overexpressing lines (OE26 includes four independent transgenic lines). Plants were grown for 7 days under +Pi (1.25 mM KH_2PO_4), then transferred to +Pi (1.25 mM KH_2PO_4) and –Pi (no Pi) conditions for 14 days as described in the materials and methods. Values are the means ± SE of three biological replicates.
